# New Appliances and Things Medical

**Published:** 1894-06-23

**Authors:** 


### NEW APPLIANCES AND THINGS MEDICAL.

[All preparations, appliances, novelties, &c., of which a notice is desired, should be sent for the Editor, to care of The Manager, 428, Strand, London, W.C.]

### DIASTASED MEAL BISCUITS.

(CALLARD AND CO., 65, REGENT STREET.)

The above firm have forwarded us a sample of their new digestive biscuits. They claim for them that they are nourishing, appetising, and easy of digestion. The biscuits are rather what we should consider a hungry man's food, and should hardly describe them as appetising, if they save the stomach they certainly take it out of the teeth. With regard to digestibility, our laboratory experiments indicate that a considerable percentage of the biscuit is already converted into sugar, and hence, after having once reached the stomach, readily absorbed. There can be no doubt that, as far as farinaceous biscuits can be digestible, these biscuits are so, but we should hesitate to recommend them to persons whose digestion has been upset, or as a food for infants or young children.

### LORETIN, A NEW ANTISEPTIC POWDER.

(BURROUGHS, WELLCOME, AND CO., LONDON, E.C.)

Loretin is a fanciful name given to a substance the chemical definition of which is meta-iodo-ortho-oxyquinoline-ana-sulphonic acid. It is a bright sulphur-yellow coloured powder, somewhat resembling iodoform in appearance but almost odourless. It is anticipated that in a large measure it will replace this drug in its application to surface and granulating wounds. It has been proved to be a powerful antiseptic in (1 in 1,000) solution. It, moreover, has the advantage of being quite free from toxic effects in the system. It has no irritating properties on the kidneys, nor on the surfaces to which it is applied. In veterinary practice it has already met with a considerable measure of success in the treatment of suppurating wounds and in eczematious condition of the skin.

